# Extramammary Paget's disease with the appearance of a nodule: a case report

**DOI:** 10.1186/1471-2407-10-405

**Published:** 2010-08-04

**Authors:** Xia Wang, Wenlin Yang, Jian Yang

**Affiliations:** 1 Department of Dermatology, the Second Affiliated Hospital of Guangzhou Medical University, Guangzhou 510260, P. R. China

## Abstract

**Background:**

Extramammary Paget's disease (EMPD) remains a rare condition with only a limited number of cases reported in the literature. EMPD is mainly composed of intraepidermal Paget cells, and possesses variable clinical behaviors and histological appearances, leading to difficulty in the diagnosis of this disease.

**Case presentation:**

We here report a case of primary EMPD with the appearance of a nodule on the background of erythema. Histological assessment showed Paget cell infiltration throughout the epidermis with dermal spread. Using immunohistochemistry, the expressions of CK7, CK19, CK20, GCDFP-15, CEA, S-100 protein and bcl-2 were examined to elucidate the cellular differentiation of the carcinoma.

**Conclusion:**

According to the histological assessment, this case was diagnosed as primary EMPD with carcinoma cells invading into the dermis, but without lymph node infiltration.

## Background

EMPD of the vulva is a rare intraepithelial adenocarcinoma which accounts for less than 1% of carcinomas in vulva, while the majority of the patients are postmenopausal Caucasian females[1-3]. The cancer cells in the neoplasm usually stay "in situ" and only rarely invade into the dermis to be metastatic via the lymphatic system [4,5]. One has to differentiate neoplasms with Paget phenomenon from carcinomas metastasized from adjacent organs such as the urinary system and rectum [6]. Here, we present an unusual case of EMPD with carcinoma cells invading into the dermis without lymph node infiltration in a Chinese woman.

## Case presentation

**Figure 1:**
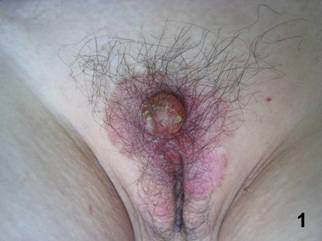
**The clinical features of the patient with EMPD**. Red firm mass on the centre of irregular warm eczematoid erythema covering the woman mons pubis.

A 66-year-old Chinese woman presented with a slowly growing vulvar mass with pruritus. Two years prior to presentation, she noted a painless red firm nodule on her vulva. Subsequently, erythema around the nodule appeared. The nodule and erythema had gradually enlarged during the 2-year period. Physical examination revealed a 2 cm × 2 cm × 2 cm slightly erosive, nodular red firm mass on the centre of 5 cm × 7 cm area of irregular eczematoid erythema covering her mons pubis (Fig.1). There was no swelling of lymph nodes in bilateral groins. Biopsy of inguinal lymph node was negative. The complete mass and part of the erythema were excised for histological evaluation. The patient's medical history was unremarkable. Metastasis from other cancers was excluded by routine blood tests, cystoscopy, oesophagogastroduodenoscopy, colonoscopy, abdominopelvic computed tomography (CT), chest radiography and positron emission tomography-CT. All these examinations were normal. At 3-month follow-up, the patient had local mass recurrence and non-palpable inguinal lymph nodes.

**Figure 2:**
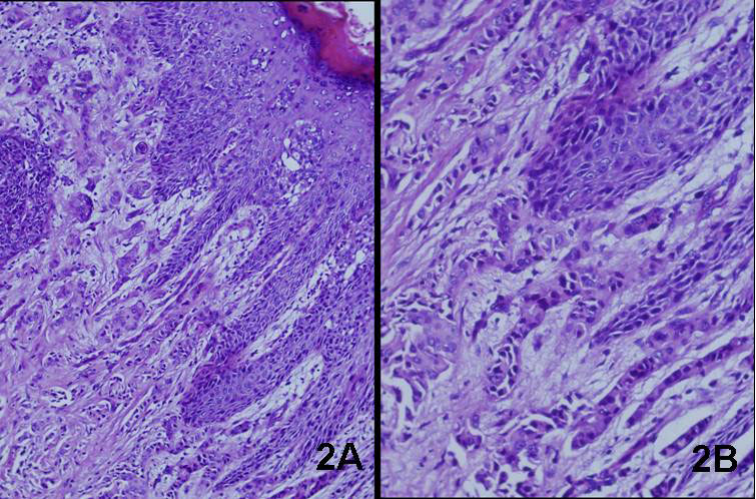
**Histological assessment of EMPD**. (A) H&E staining indicated Paget cells into the epidermis (original magnification × 100), and (B) The dermis contained a poorly differentiated adenocarcinoma (original magnification × 200).

**Figure 3:**
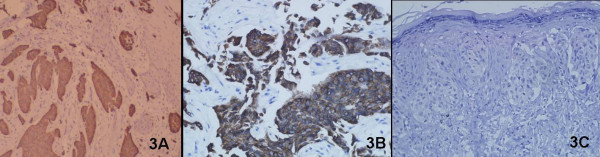
**Immunohistochemical assessment of EMPD**. (A) The carcinoma showed positivity for GCDFP-15(original magnification × 100).(B) The carcinoma showed positivity for CK7 (original magnification × 200).(C) The carcinoma showed negative for CK20 (original magnification × 200)

Hematoxylin and Eosin staining of the biopsy from the lesion showed large round cells with ample pale-staining cytoplasm, pleomorphic nuclei, and occasional prominent nucleoli infiltrating throughout the epidermis, indicative of Paget cells (Fig. 2A). This carcinoma spread dermal, and a poorly differentiated adenocarcinoma was present in the dermis.(Fig. 2B). Immunohistochemical stainings for gross cystic disease fluid protein-15 (GCDFP-15) (Fig. 3A), cytokeratin7(CK7) (Fig. 3B), cytokeratin 19 (CK19) and carcinoembryonic antigen (CEA) were strongly positive. Epithelial membrane antigen (EMA) was positive, while S-100 protein, bcl-2 and CK20 (Fig. 3C) were all negative. Neoplastic cells were positive for periodic acid-Schiff (PAS). Moreover, the Paget cells and low-differentiated adenocarcinoma shared common immunohistological features. These immunohistochemical appearances supported the diagnosis of EMPD.

## Discussion

EMPD is an intraepidermal adenocarcinoma, which usually develops in locations with apocrine glands. Nevertheless, when the carcinoma cells infiltrate into the dermis and form a nodule, this disease turns into an invasive carcinoma belonging to adnexal adenocarcinoma of the skin [7]. Typically involved anatomical sites are the vulvar, perianal, perineal, scrotal and penile regions. Clinically, the lesions present as well-defined, moist, erythematous plaques usually accompanied by pruritus. The study reported by Hatta et al [8] revealed that erythema was a clinical characteristic of all lesions; furthermore, nodules were present in 24%, erosion in 49% and hypopigmentation in 25% cases. In addition, 39% of patients exhibited lymphopathy.

Primary adenocarcinomas of the vulva have been classified into sweat gland cancers, EMPD [9,10], and"breast-like''adenocarcinomas of the vulva [11,12]. Histologically, sweat gland carcinomas of the vulva possess adenopapillary cords and tubules, occasionally comprising pagetoid cells. Although EMPD mainly consists of intraepidermal Paget cells, dermal invasion with cords and sheets has also been recorded [13]. The current opinion is that the malignancy spreads from superficial to deep [14], rather than from deep to superficial [13,15]. Primary breast carcinoma of the vulva exhibits histological characteristics similar to breast carcinoma. These three carcinomas may possess some consistent histopathological features. Van der Putte and van Gorp [16] put forward the term "adenocarcinoma of the mammary-like glands of the vulva", leading to a novel unifying notion for the three diseases mentioned above.

The level of invasion of the paget cells in EMPD can be classified into three grades: in situ in the epidermis, microinvasion to the papillary dermis, and deep invasion into the reticular dermis or subcutaneous tissue [17]. According to the above classification method, this case can fall into the third grade. Hatta et al [8] reported that male patients outnumbered female patients (male-female ratio, 2.6 to 1) in Japanese, in contrast to previous reports from western countries. The discrepancy between the studies can be explained by genetic variety of different regions. Further epidemiological studies for this disease are needed to clarify this point.

In this case, the lesion was present as a painless, slowly-growing, red firm mass on the centre of irregular eczematoid erythema covering the elderly woman's mons pubis. Histological assessment showed EMPD with carcinoma cells invading into the dermis, which are often characteristics of the metastatic lesion of breast. However, metastasis from breast carcinoma and internal malignancies was excluded by physical and auxiliary examinations. When carcinoma cells infiltrate into the dermis and advance to Paget's carcinoma, it is referred to as adenocarcinoma of the skin and has a poor prognosis. Up to now, no established guideline has been made for the diagnosis of EMPD, the nonspecific clinical findings of EMPD often lead to misdiagnosis and extended periods of topical and systemic medical mismanagement.

Because the disease is rare, there is little knowledge of the most effective treatment. Primary treatment is surgical and involves wide local excision with frozen section evaluation of margins. Invasion level and multiple lymph node metastases are important prognostic factors in EMPD. Unfortunately, there is a high rate of recurrence. Some suggest prophylactic regional lymph node dissection, especially for high grade carcinomas, whereas others recommend removal of only clinically involved nodes [9]. Reports on the results of radiotherapy are conflicting: these carcinomas are radioresistant, or radiotherapy increases local control [3]. Radiation therapy may be used as a supplement to aggressive surgery. We recommend that treatment for EMPD includes surgery in the form of wide local excision and adjuvant radiotherapy with caution and individualization.

## Conclusions

Primary EMPD of the vulva is exceptional. Up to now, no gold diagnostic standard has been established for EMPD, leading to the difficulty in diagnosis of this disease. To improve its prognosis, further investigation needs to be performed for the early detection of primary EMPD and its metastasis.

## Consent

Written informed consent was obtained from the patient for publication of this case report and any accompanying images. A copy of the written consent is available for review by the Editor-in-Chief of this journal.

## Abbreviations

EMPD: Extramammary Paget's disease; CT: computed tomography; GCDFP-15: gross cystic disease fluid protein-15; CK7: cytokeratin 7; CK19: cytokeratin 19; CK20: cytokeratin 20; CEA: carcinoembryonic antigen; EMA: epithelial membrane antigen; bcl-2: b cell lymphoma/lewkmia-2; PAS: periodic acid-schiff

## Competing interests

The authors declare that they have no competing interests.

## Authors' contributions

XW drafted the manuscript and in the data collection. WLY participated in the design and coordination of the case-study and revised the manuscript for important intellectual content. JY carried out the histological examination and diagnosed, investigated, followed up and managed the patient. All authors have read and approved the final manuscript.

## Authors' information

**Xia Wang**, Resident Doctor, Department of Dermatology, the Second Affiliated Hospital of Guangzhou Medical University, Guangzhou 510260, P. R. China. E-mail: wxpiglet@yahoo.com.cn.

**WenLin Yang**, Professor, Department of Dermatology, the Second Affiliated Hospital of Guangzhou Medical University, Guangzhou 510260, P. R. China. E-mail: wenliny@21cn.com.

**Jian Yang**, Professor, Department of Dermatology, the Second Affiliated Hospital of Guangzhou Medical University, Guangzhou 510260, P. R. China. E-mail: Yangj123@21cn.com.

## Pre-publication history

The pre-publication history for this paper can be accessed here:

http://www.biomedcentral.com/1471-2407/10/405/prepub
